# A case report of autoimmune pancreatitis associated with a pancreatic pseudocyst

**DOI:** 10.1097/MD.0000000000010439

**Published:** 2018-05-11

**Authors:** Kai Zhang, Xianying Liu, Lei Yi, Jiannan Li, Jian Shi, Tongjun Liu

**Affiliations:** a Department of General Surgery; b Medical Department, The Second Hospital of Jilin University, Changchun, Jilin, China.

**Keywords:** autoimmune pancreatitis, distal pancreatectomy, pseudocyst

## Abstract

**Rationale:**

Autoimmune pancreatitis (AIP) is a special type of chronic pancreatitis, which is rarely associated with pseudocyst.

**Patient concerns:**

A 48-year-old man complained of a recurrent upper abdominal pain in our hospital.

**Diagnoses:**

A cystic mass of size 4 × 3 cm in his pancreatic tail was found by computed tomography. The concentrations of serum carbohydrate antigen19–9 (81 U/mL) and serum immunoglobulin G4 (181 mg/dL) were elevated.

**Interventions:**

The patient received partial pancreatectomy with splenectomy and partial esophagectomy.

**Outcomes:**

Further histopathological examination revealed a pseudocyst, significant lymphoplasmatic infiltration, and fibrosis in the pancreas and esophagus. We report a rare case of AIP complicated with a pancreatic pseudocyst and invasion of lower esophagus.

**Lessons:**

Our study demonstrated that surgical therapy should be considered for the refractory AIP complicated with a pancreatic pseudocyst and invasion of lower esophagus.

## 1 Introduction

Autoimmune pancreatitis (AIP) is a special type of chronic pancreatitis, which has a diagnostic criterion defined by the International Association of Pancreatology.^[1]^ AIP is recognized as an IgG4-related pancreatic disease, which frequently shows an elevated level of IgG4.^[2]^ Tough AIP is rare, it is presented with pseudocyst like other pancreatitis. However, controversies exist on treatment strategy for AIP associated with pseudocyst. Here, we report a rare case which was clinically diagnosed with AIP complicated with a pancreatic pseudocyst and invasion of lower esophagus, the patient was resistant to steroid therapy, and underwent surgical treatment.

## 2 Case report

This study was approved by the Ethics Committee and institutional review board of the Second Hospital of Jilin University, Changchun, China. Written informed consent was obtained from the patient for publication of this report.

**Figure 1:**
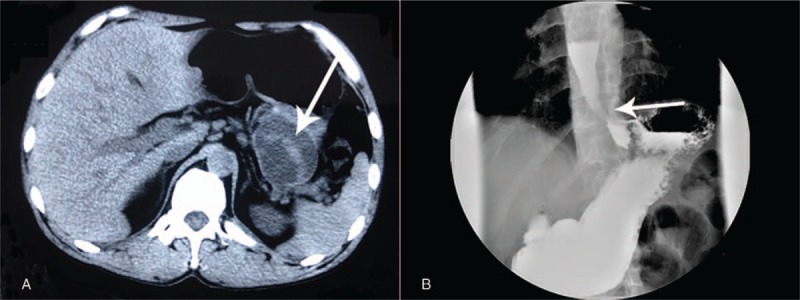
Diagnostic computed tomography (CT) and upper gastrointestinal radiography. (A) CT obviously enlarged cystic mass of pancreas, (B) Esophageal stenosis (white arrowhead).

**Figure 2:**
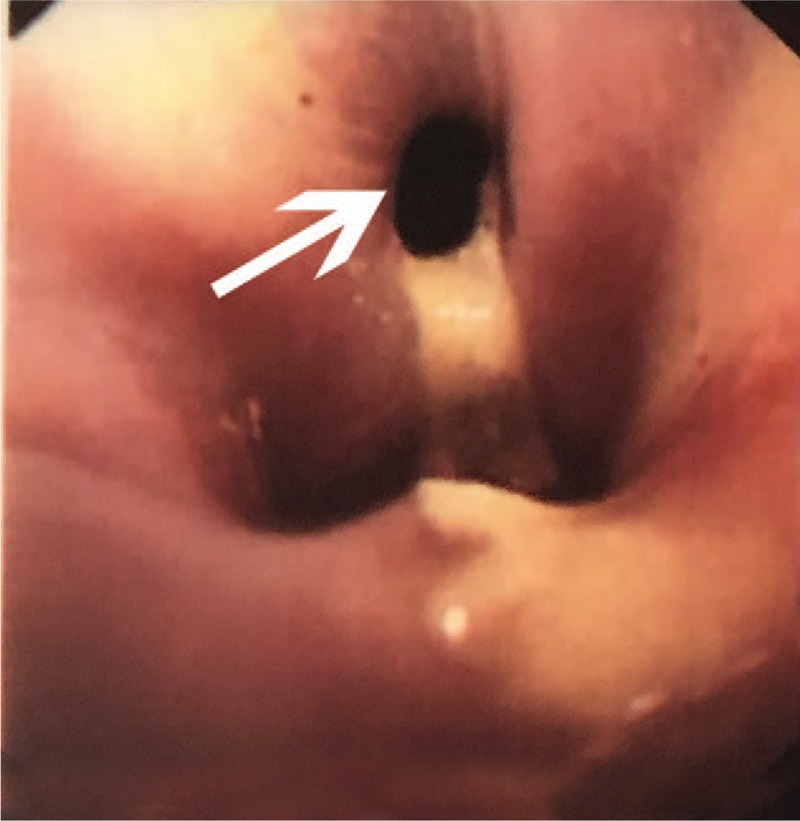
Fistula in esophagus by gastroscopy (white arrowhead).

**Figure 3:**
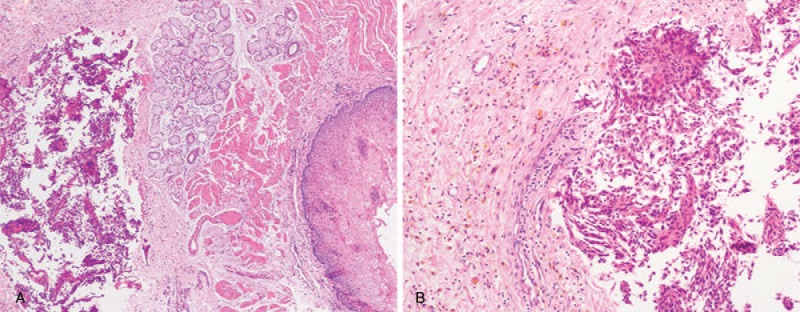
HE staining of mass. (A) Significant lymphoplasmatic infiltration and fibrosis in the pancreas, (B) and significant lymphoplasmatic infiltration and fibrosis in esophagus.

A 48-year-old man went to another hospital because of upper abdominal pain. AIP was diagnosed, and the pain resolved after standard steroid therapy. The patient received prednisone therapy with 30 mg/day, tapering by 5 mg every week for 2 months. The patient's condition was in remission without any progressive symptoms or signs. However, 3 months later, he was admitted to our hospital because of recurrent upper abdominal pain. Physical examination showed no mass was palpable on abdomen and no jaundice was found. He had no history of smoking or drinking. Auxiliary examinations revealed a cystic mass in the pancreas with a mild elevated concentrations of serum carbohydrate antigen19–9 (CA19–9) (81 U/mL) and serum immunoglobulin G4 (IgG4) (181 mg/dL). Serum and urine amylase levels were in normal range. A cystic mass of size 4 × 3 cm in his pancreatic tail was found by computed tomography (CT) (Fig. 1A). Furthermore, distal esophageal stenosis was found by upper gastrointestinal radiography (Fig. 1B) and fistula in esophagus was found by gastroscopy (Fig. 2). In consideration of these information and his medical history, he was diagnosed with AIP complicated with a pseudocyst and distal esophageal stenosis. Because of his poor response to steroid therapy, suspicion of cystic neoplasm, and invasion of lower esophagus, the patient underwent distal pancreatectomy with splenectomy and partial esophagectomy. The surgery was successful; the patient underwent an uneventful postoperative course and discharged from hospital after 10 days. The postoperative serum concentration of CA19–9 decreased to 21 U/L, levels of serum CA19–9 (25 U/L) and serum IgG4 (38 mg/dL) were decreased significantly. Hematoxylin-eosin staining revealed a pseudocyst, significant lymphoplasmatic infiltration, and fibrosis in the pancreas (Fig. 3A) and esophagus (Fig. 3B).

In the period of 6 months of follow-up, the patient did not take prednisone because the serum concentrations of CA19–9 and IgG4 returned to normal, and the patient's condition was in complete remission without any progressive symptoms.

## 3 Discussion

Pancreatic cyst formation in AIP may be related to a highly active state of the inflammatory process^[3]^ and severe narrowing of the branched pancreatic ducts.^[4]^ Steroids are considered as inducing pseudocyst regression via inhibition of inflammation of the pancreatic duct.^[5]^ Patients with AIP show an excellent response to steroid therapy; however, it has been reported that AIP relapse rate is 92% within 3 years,^[6]^ suggesting further treatment options are needed. Surgical treatment can be an effective option for the patients of ineffective steroid therapy. AIP patients who were resistant to steroid therapy could undergo total pancreatectomy with islet autotransplantation for symptom relief.^[7]^ We believe that partial pancreatectomy with splenectomy is another alternative option for AIP complicated with pseudocyst when the patient is insensitive to steroid treatment and with suspicion of malignancy.

Here, we present a case of AIP with a pancreatic pseudocyst and esophageal stenosis without good response to steroid therapy treated by partial pancreatectomy with splenectomy and partial esophagectomy. In this case, the cystic lesion in the pancreas was found after standard steroid treatment. The serum IgG4 and CA19–9 concentration increases. Upper gastrointestinal radiography revealed esophageal stenosis. For poor response to steroids of the cystic mass, and the suspicion of malignancy, distal pancreatectomy with splenectomy was chosen for this patient. During postoperative follow-up, serum concentrations of IgG4 and CA19–9 returned to normal, but further long-term follow-up of serum IgG4 levels and CT investigation should be considered in case of recurrence of AIP or pseudocyst.

In conclusion, in our described case, surgical resection was the most effective method of treatment and it should be considered as initial treatment in such cases.

## Author contributions

**Conceptualization:** Jian Shi.

**Formal analysis:** Xianying Liu.

**Funding acquisition:** Xianying Liu.

**Investigation:** Tongjun Liu.

**Project administration:** Lei Yi.

**Writing – original draft:** Kai Zhang.

**Writing – review & editing:** Kai Zhang.
